# The importance of early referral for the treatment of chronic kidney disease: a Danish nationwide cohort study

**DOI:** 10.1186/1471-2369-13-108

**Published:** 2012-09-10

**Authors:** Kristine Hommel, Mette Madsen, Anne-Lise Kamper

**Affiliations:** 1 Department of Nephrology, Rigshospitalet, Copenhagen University Hospital, Blegdamsvej 9, Copenhagen East, 2100, Denmark; 2 National Institute of Public Health, University of Southern Denmark, Øster Farimagsgade 5A, Copenhagen K, 1353, Denmark; 3 Department of Public Health, University of Copenhagen, Øster Farimagsgade 5, Copenhagen K, 1014, Denmark

**Keywords:** Chronic kidney failure, Epidemiology, Late diagnosis, Treatment, Renal replacement therapy

## Abstract

**Background:**

Many patients with advanced chronic kidney disease are referred late to renal units. This is associated with negative aspects. The purpose of the present study was to characterize late versus early referrals for renal replacement therapy including their renal disease, health care contacts and medical treatment before renal replacement therapy (RRT) and the consequences for RRT modality and mortality.

**Methods:**

Nationwide cohort study including 4495 RRT patients identified in the Danish Nephrology Registry 1999–2006. The cohort was followed to end 2007 by linkage to other national registries. Late referral: follow-up ≤16 weeks in renal unit before RRT start. Cox proportional hazards models were used to estimate the relative risk of mortality or waiting list status within 365 days in late referrals versus early referrals.

**Results:**

A total of 1727 (38%) incident RRT patients were referred late. Among these, 72% were treated in non-nephrology hospital departments and 91% in general practice 2 years to 16 weeks before RRT start. Fewer late referrals received recommended pre-RRT treatment as judged by renin-angiotensin-system blockade: 32% versus 57% or the D-vitamin analogue alfacalcidol: 5% versus 30% (P < .001). Primary RRT modality was peritoneal dialysis: 18% in late versus 32% in early referrals (P < .001), 7% versus 30%, respectively, had an arteriovenous dialysis-fistula (P < .001) and 0.2% versus 6% were on the waiting-list for renal transplantation (P < .001) before RRT start. One-year-mortality was higher in late referrals: hazard ratio 1.55 (CI 95% 1.35–1.78). In a subgroup, 30% (CI 95% 25–35%) late and 9% (CI 95% 6–12%) early referrals had plasma creatinine ≤150% of upper reference limit within 1 to 2 years before RRT start (P < .001).

**Conclusions:**

Late nephrology referrals were well-known to the healthcare system before referral for RRT start and more often had near normal plasma creatinine levels within 2 years before RRT start. They infrequently received available treatment or optimal first RRT modality. An increased effort to identify these patients in the healthcare system in time for proper pre-dialysis care including preparation for RRT is needed.

## Background

Treatment of advanced chronic kidney disease (CKD) includes renoprotective therapy, prevention of CKD-related complications and preparing for chronic renal replacement therapy (RRT). It is well-known that late referral of CKD patients to renal units is associated with several negative aspects such as increased mortality; although many studies are limited by a lack of data on the history of CKD progression in individuals before RRT start [1-12]. However, late referral still remains a major problem.

To establish a strategy on earlier referral it is important to get information on patients referred late, including their renal disease progression and previous contacts to the health care system. The aim of the present study was to characterize late versus early CKD referrals, their contacts to the health care system before RRT start and consequences for the treatment. It was a nationwide study offering complete follow-up.

## Methods

The Danish population consisted of 5.3 million persons in 1999 increasing to 5.4 in 2006 [13]. Denmark has a tax-financed universal health care system with free access for all citizens to general practitioners, hospitals and essential operations. During the study period, the number of renal units was 15. No private dialysis centre’s exist.

### Patients

The study population consisted of all 5513 incident chronic RRT patients in 1999–2006 in Denmark, except patients from Greenland and the Faroe Islands or patients without residence permit. Cross-linkage between registries was made with the unique personal identification number assigned to all Danish citizens from birth or immigration.

### Data sources

Information on date of chronic RRT start, modality, renal diagnosis and date of death were obtained from the Danish Nephrology Registry [14], where all patients actively treated for end-stage CKD are registered. Only patients with at least 3 months need of RRT are included in the registry, thereby excluding patients with acute reversible renal failure.

Information on courses in hospital was obtained from the National Patient Registry [15]. This administrative registry contains information on all hospital admissions in Denmark including diagnoses, operations, dates, hospitals and departments since 1977 and from 1995 also information on out-patient treatment. It is mandatory for all hospital departments to report to the registry as reimbursement is based on this information [16,17].

Information on contacts to general practice was obtained from the Danish National Health Service Registry. This administrative registry contains information on all contacts to and services given by general practitioners since 1990 [18].

Information on medical treatment was obtained from the Register of Medicinal Product Statistics of the Danish Medicines Agency. The registry contains information on prescribed and sold drugs since 1994 excluding in-hospital drug use [19].

Information on all patients listed for renal transplantation since 1995 was obtained from the Scandia transplant database [20].

Individual level information on income, highest level of completed education, immigration and ethnic origin was obtained from Statistics Denmark [13].

Level of renal function before RRT start was evaluated in the subgroup of patients with residence in Copenhagen Municipality 2001–6. Information on plasma creatinine was obtained from databases of the departments of clinical biochemistry in three of four hospitals in Copenhagen and the Laboratory of the General Practitioners in Copenhagen. Data from one hospital was lost with a local change of software. Not all laboratories used plasma creatinine measurement methods standardized or traceable to isotope dilution mass spectrometry (IDMS). Therefore we could not calculate valid estimated glomerular filtration rates with the MDRD [21] study formula. Instead patients were classified according to plasma creatinine level 1–2 years before RRT start: i) plasma creatinine >150% of upper reference limit, ii) plasma creatinine ≤150% of upper reference limit or iii) unknown plasma creatinine.

### Definitions

Late referral of patients with CKD was defined as a course in a renal unit starting within 16 weeks or less before RRT start and *early referral* accordingly as courses starting more than 16 weeks before. Patients who had had a previous course in a renal unit ending more than 2 years before RRT start were classified as late referrals.

In and outpatient courses in any non-nephrology department were studied two years to 16 weeks before RRT start. Some patients had contact to more than one specialty. Courses in cardiology and endocrinology were contained within contacts to departments of internal medicine and equally contacts to urology departments within contacts to any surgical department.

Contacts to general practice were studied two years to 16 weeks before RRT start and patients were classified as: i) patients seen in the clinic, ii) contacts to general practitioner by e-mail or telephone only or iii) no contact to general practitioner.

Pre-RRT renal care was evaluated by medical treatment with renin-angiotensin system (RAS) blocking agents, the vitamin-D analogue alfacalcidol and NSAID judged by prescriptions filled within the period two years to 16 weeks before RRT start. Values of defined daily dose (DDD) were made by the WHO based on an average dose per day for an adult using the drug for the main indication [22]. A minimum of 1:3 of the time (206 days) covered by at least 0.25 μg of alfacalcidol, 1:3 of the time (206 days) covered by a mean of ½ DDD of RAS inhibitor or at least 16% of the time (100 days) covered with a mean of one DDD of NSAID defined the patients treated. One DDD of RAS inhibitor alone or in combination with other drugs equals 10 mg enalapril or 50 mg captopril. One DDD of NSAID equals 1200 mg of ibuprofen.

Comorbidity was evaluated within 2 years before RRT start by ICD-10 diagnose of in and for cancer also outpatient hospital course: i) acute myocardial infarction; I22-22, ii) cerebrovascular disease: I60-I64, iii) cancer (excluding non-melanoma skin cancers): C00-43 and C45-C99 and iiii) bacteraemia: A41-42. Furthermore mean number of inpatient hospital-days and number of visits in general practice within 2 years before RRT start was used as a measure of comobidity.

Income was defined retrospectively as total income in the year 5 years before start of RRT, corrected for inflation according to the price index in Statistics Denmark and grouped into low -, medium- and high corresponding to the level of approximate tertiles in the Danish 2006 population aged 30 to 69 years.

Patients were divided into 3 groups according to the highest level of completed education: i) primary school, ii) high school and skilled craftsmen and iii) persons with university degrees, nurses, librarians, school teachers etc.

Ethnic origin was defined according to the patients’ and their parents’ country of birth and citizenship [13]. Origin was divided into Danish and for immigrants and descendants into western and non-western [23].

Details on definitions of education, income and ethnic origin are described elsewhere [24].

### Statistics

Chi-squared, Fisher’s exact and Wilcoxon signed rank sum test were used where appropriate. Logistic regression was used to compare age, sex, income, education and renal diagnosis in late referrals versus early referrals. Likelihood ratio test for interaction was made for confounder and exposure variables: age, sex, income, education, renal diagnosis and referral. Type 3 test was used to test the effect of income and education. Cox proportional hazards models, with time to death or renal transplantation waiting list status as outcome variables, were used to estimate the relative risk of mortality or waiting list status within 365 days in late referrals versus early referrals. Adjustment was made for differences in age, sex, renal diagnoses, comorbidity and emigration. The proportional hazards assumption was met. All variables were kept in the models regardless of significance level. Analyses were performed using SAS version 9.2, SAS Institute Inc.

### Ethical considerations

The study was approved by the Danish Data Protection Agency. Data was anonymised to ensure privacy of the involved patients.

## Results

Patients from 5 of 15 renal units were excluded because of non-specific classification of hospital departments to the NPR. These included 886 patients (16%) of the total 5513 incident RRT patients. A total of 86 patients (2%) were excluded because of renal diagnoses indicating irreversible acute renal failure: haemolytic uremic syndrome and acute tubular necrosis or age less than one year. Also, 46 patients (1%) who immigrated within the 2 years before RRT start were excluded. Basic characteristics of the patients included and excluded regarding sex, age and renal diagnoses were similar. The remaining cohort was 4495 patients.

In a total of 4495 patients, 1727 (38%) were referred late to a renal unit before RRT start and 806 patients (18%) very late, within 1 week (Table 1).

**Table 1:** Characteristics of late versus early nephrology referrals before renal replacement therapy start

	**Late referrals**^**a**^	**Early referrals**^**b**^	**P value**
Incident RRT patients, n	1727	2768	
Age, median (IQR)	67 (55–75)	63 (52–73)	<.001
Women, n (%)	653 (38%)	1024 (37%)	.58
Age women, median (IQR)	68 (54–75)	64 (51–73)	<.001
Patients aged ≥70 years, n (%)	720 (42%)	905 (33%)	<.001
Women in patients aged ≥70, n (%)	278 (39%)	340 (38%)	.67
Ethnic origin non-western countries^c^, n (%)	65 (4%)	132 (5%)	.11
Ethnic origin western countries^d^, n (%)	43 (2%)	68 (2%)	.94
Diabetic end-stage renal disease^e^, n (%)	348 (20%)	706 (26%)	<.001
Adult polycystic kidney disease^f^, n (%)	57 (3%)	261 (9%)	<.001
Chronic glomerulonephritis^g^, n (%)	138 (8%)	346 (13%)	<.001
Comorbidity			
Acute myocardial infarction^h^, n (%)	99 (6%)	119 (4%)	.03
Stroke^i^, n (%)	81 (5%)	81 (3%)	.002
Cancer in total^j^, n (%)	216 (13%)	156 (6%)	<.001
Cancer in the urinary tract^k^, n (%)	49 (3%)	34 (1%)	<.001
Genital cancer^l^, n (%)	41 (2%)	43 (2%)	.05
Bacteraemia^m^, n (%)	48 (3%)	62 (2%)	.25
Days in hospital, median^n^ (IQR)	28 (14–52)	16 (5–37)	<.001
Visits in general practice, median^n^ (IQR)	11 (5–20)	10 (5–19)	.08
Patients 30–69 years, n	924	1748	
Primary school only, n (%)	420 (45%)	731 (42%)	.07
Low income^o^, n (%)	566 (61%)	1001 (57%)	.05

Abbreviations: RRT, renal replacement therapy; IQR, interquartile range.

^a^Late referrals: Course in a nephrology department ≤16 weeks.

^b^Early referrals: Course in a nephrology department >16 weeks.

^c^Western countries: Andorra, Australia, Canada, EU, Iceland, Monaco, New Zealand, Norway, San Marino, Switzerland, USA and the Vatican State.

^d^Non-western countries: those not being western countries or Denmark.

^e^Diabetic end-stage renal disease (ICD-10 E10–E14).

^f^Adult polycystic kidney disease (ICD-10 Q61).

^g^Chronic glomerulonephritis (ICD-10 N00–N08).

^h^Admitted to hospital within 2 years before RRT start with ICD-10 diagnoses: I21 or I22.

^i^Admitted to hospital within 2 years before RRT start with ICD-10 diagnoses: I60, I61, I62, I63 or I64.

^j^Admitted to hospital or course in outpatient clinic within 2 years before RRT start with ICD-10 diagnoses: C00–C43 or C44–C99.

^k^Admitted to hospital or course in outpatient clinic within 2 years before RRT start with ICD-10 diagnoses: C64–68.

^l^Admitted to hospital or course in outpatient clinic within 2 years before RRT start with ICD-10 diagnoses: C51–58 or C61–63.

^m^Admitted to hospital within 2 years before RRT start with ICD-10 diagnoses: A41–42.

^n^Within 2 years before RRT start.

^o^Low income: ≤30,200 € per year (33.7 % of the Danes with the lowest income 2006, corrected for inflation).

Median age of late referrals and the proportion of patients aged 70 years or more were higher in late compared to early referrals. When adjusting for sex and renal diagnosis, odds ratio (OR) for late referral was 1.29 in patients aged ≥70 years compared to <70 years (P < .001) (Table 2). The proportion of women was stable 37–39% in total and among the 70 year olds in early and late referrals. Patients with the common renal diagnoses diabetic nephropathy, adult polycystic kidney disease or chronic glomerulonephritis were more frequently early referrals. When adjusting for differences in age and sex, OR for late referral in patients with diabetic nephropathy was 0.62, chronic glomerulonephritis 0.52 and polycystic kidney disease 0.27 (P < .001) compared to the rest of the patients. There was a significant interaction between age and diabetes as old diabetic patients had a similar risk of late and early referral. Referral pattern was similar in patients aged 30–69 years regardless of length of education (P = .14) and level of income (P = .06).

**Table 2:** Risk of late nephrology referral before start of renal replacement therapy

	**OR (95 % CI)**	**P value**
Patients ≥70 years^a^	1.47 (1.30–1.67)	<.001
Patients ≥70 years^b^	1.29 (1.13–1.47)	<.001
Diabetic nephropathy^c^	0.62 (0.53–0.72)	<.001
Diabetic nephropathy ≥70 years^d^	0.81 (0.63–1.05)	.12
Diabetic nephropathy <70 years^e^	0.54 (0.45–0.66)	<.001
Chronic glomerulonephritis^c^	0.52 (0.42–0.64)	<.001
Adult polycystic kidney disease^c^	0.27 (0.20–0.37)	<.001
Medium / short education^f^	0.93 (0.78–1.12)	.45
Long / short education^f^	0.88 (0.67–1.16)	.36
Medium / low income^g^	0.99 (0.81–1.21)	.94
High / low income^g^	0.77 (0.58–1.01)	.06

Abbreviations: Late referral, course in a nephrology department ≤16 weeks; OR, odds ratio; 95 % CI, 95 % confidence interval.

^a^Reference group: patients <70 years, adjusted for sex.

^b^Reference group: patients <70 years, adjusted for sex and renal diagnoses.

^c^Reference group: patients with other renal diagnoses than diabetic. nephropathy, chronic glomerulonephritis or adult polycystic kidney disease, adjusted for sex and age.

^d^Reference group: patients ≥70 years with other renal diagnoses than diabetic nephropathy, chronic glomerulonephritis or adult polycystic kidney disease, adjusted for sex.

^e^Reference group: patients <70 years with renal diagnoses other than diabetic nephropathy, chronic glomerulonephritis or adult polycystic kidney disease, adjusted for sex.

^f^Reference group: patients with short education, adjusted for age, sex, renal diagnoses and income.

^g^Reference group: patients with low income, adjusted for age, sex, renal diagnoses and education.

Late referrals had more often been admitted to hospital within 2 years before RRT start because of acute myocardial infarction, stroke or cancer. Also, late referrals had more days in hospital compared to early referrals. There was no difference between the two referral groups in the frequency of bacteraemia.

Among late referrals, 1247 (72%) had had a course in a non-nephrology department within 2 years to 16 weeks before RRT start (Table 3). These included departments of endocrinology: 116 patients (7%), cardiology: 160 (9%) and urology: 121 (7%). The proportion of patients with a course in a non-nephrology department was lower (P < .001) in late compared to early referrals. A total of 119 patients (7%) among late referrals had had a course in a nephrology department which terminated more than two years before RRT start. Totally 1567 late referrals, (91%) were seen in general practice and 1047 (61%) had had blood tests done.

**Table 3:** Hospital courses and contacts to general practice according to early or late nephrology referral

**Courses 2 years to 16 weeks before RRT start**	**Late referrals**^**a**^**n = 1727**	**Early referrals**^**b**^**n = 2768**	**P value**
Any non-nephrology department, n (%)	1247 (72%)	2413 (87%)	<.001
Endocrinology department, n (%)	116 (7%)	385 (14%)	<.001
Cardiology department, n (%)	160 (9%)	506 (18%)	<.001
Any department of internal medicine, n (%)	893 (52%)	1854 (67%)	<.001
Urology department, n (%)	121 (7%)	447 (16%)	<.001
Any surgical department, n (%)	902 (52%)	1849 (67%)	<.001
Nephrology department, course terminated 2 years before RRT start, n (%)	119 (7%)		
Seen by general practitioner, n (%)	1567 (91%)	2633 (95%)	<.001
Telephone/e-mail contacts only to general practice, n (%)	54 (3%)	63 (2%)	.08
Not seen by general practitioner, n (%)	106 (6%)	72 (3%)	<.001
Blood tests done in general practice, n (%)	1047 (61%)	1736 (63%)	.16
Seen in general practice or non-nephrology department	1605 (93%)	2730 (99%)	<.001

Abbreviations: RRT, renal replacement therapy; IQR, interquartile range.

^a^Late referrals: Course in a nephrology department ≤16 weeks.

^b^Early referrals: Course in a nephrology department >16 weeks.

Prescriptions of alfacalcidol or RAS blocking agents were filled by fewer late compared to early referrals (Table 4). While there was no difference between late and early referrals filling ≥1 prescription of NSAID, fewer late compared to early were treated with NSAID ≥100 days within 2 years to 16 weeks before RRT start (P < .001).

**Table 4:** Medical treatment according to late and early nephrology referral

**Prescriptions filled 2 years to 16 weeks before RRT start**	**Late referrals**^**a**^**n=1727**	**Early referrals**^**b**^**n=2768**	**P value**
≥1 prescription of alfacalcidol, n (%)	100 (6%)	1129 (41%)	<.001
Patients treated with alfacalcidol^c^, n (%)	82 (5%)	841 (30%)	<.001
≥1 prescription of RAS blocking agents, n (%)	636 (37%)	1792 (65%)	<.001
Patients treated with RAS blocking agents ^d^, n (%)	548 (32%)	1567 (57%)	<.001
≥1 prescription of NSAIDs, n (%)	485 (28%)	712 (26%)	.08
Patients treated with NSAIDs^e^, n (%)	166 (10%)	188 (7%)	<.001

Abbreviations: RRT, renal replacement therapy; RAS, renin-angiotensin system; DDD, defined daily dose.

^a^Late referrals: Course in a nephrology department ≤ 16 weeks.

^b^Early referrals: Course in a nephrology department > 16 weeks.

^c^A minimum of 1:3 of the time (206 days) covered by a mean of 0.25 μg of alfacalcidol.

^d^A minimum of 1:3 of the time (206 days) covered by a mean of ½ DDD of RAS inhibitor.

^e^A minimum of 16 % of the time (100 days) covered with a mean of one DDD of NSAID.

In total, 314 (18%) late compared to 897 (32%) early referrals started RRT with peritoneal dialysis (Table 5). A lower proportion of late compared to early referrals had an arteriovenous vascular access before RRT start. Fewer late (8%) compared to early (21%) referrals were on the waiting-list for a renal transplant within one year after RRT start. Few patients died while waiting. One-year renal transplantation waiting-list status differed with a hazard ratio of 0.32 (CI 95% 0.26–0.38) in late compared to early referrals when adjusted for differences of age, sex, renal transplantation within one year, death, emigration, comorbidity and renal diagnoses. Median number of days in hospital 1–60 days after RRT start was higher (P < .001) in late: 16 days (interquartile range 6–30) compared to early referrals: 4 days (interquartile range 0–15).

**Table 5:** Dialysis modality, access and renal transplantation waiting list status in late and early referralsw

	^**a**^**Late referrals n = 1727**	^**b**^**Early referrals n = 2768**	**P value**
Peritoneal dialysis as first RRT modality, n (%)	314 (18%)	897 (32%)	<.001
Haemodialysis as first RRT modality, n (%)	1403 (81%)	1743 (63%)	<.001
Arteriovenous vascular access before RRT start, n (%)	118 (7%)	841 (30%)	<.001
On transplantation waiting-list before RRT start, n (%)	4 (0.2%)	177 (6%)	<.001
Transplanted within one year after RRT start, n (%)	63 (4%)	279 (10%)	<.001
On transplantation waiting-list within one year after RRT start, n (%)	144 (8%)	582 (21%)	<.001
Death on the waiting-list within one year after RRT start, n (%)	2 (0.1%)	8 (0.3%)	.33

Abbreviations: RRT, renal replacement therapy.

^a^Late referrals: Course in a nephrology department ≤16 weeks.

^b^Early referrals: Course in a nephrology department >16 weeks.

A total of 435 (25%) in late referrals died within one year after RRT start compared to 412 (15%) in early referrals (P < .001). Mortality within 1 year also differed with a hazard ratio of 1.55 (95% CI 1.35–1.78) in late compared to early referrals when adjusted for differences of age, sex, emigration, comorbidity and renal diagnoses.

In a total of 370 patients, 114 (31% CI 95% 26–36%) late referrals, with residence in Copenhagen Municipality commenced RRT in 2001–6. In 33 (29% CI 95% 24–34%) of late and 206 (80% CI 95% 76–84%) of early referrals plasma creatinine was >150% of upper reference limit 1–2 years before RRT start (P < .001). In 34 (30% CI 95% 25–35%) of late and 22 (9% CI 95% 6–12%) of early referrals plasma creatinine was ≤150% of upper reference limit (P < .001). While, in 47 (41% CI 95% 36–46%) of late and 28 (11% CI 95% 25–31) of early referrals we have no information on plasma creatinine 1–2 years before RRT start (P < .001).

## Discussion

The present Danish nationwide study, including 4495 incident RRT patients in the period 1999–2006 shows that 38% of the patients had been referred to a renal unit within 16 weeks before RRT start. This is in accordance with previous reports in smaller studies reporting late referral in 22–40% of incident RRT patients when defined as a nephrology course starting ≤ 4 months before RRT start [1,2,6,7,9,10,12]. The most important finding in our study is that late referrals were well-known in the healthcare system before RRT start and yet received insufficient pre-RRT renal care.

The reasons for late referral of well-known patients are not clarified. One explanation might be fast progression of renal disease. Most studies on late referral are limited by lack of data on the history of CKD in individuals before RRT start. In the present study, a subgroup analysis in 370 patients showed that 30% of late referrals had plasma creatinine ≤150% of upper reference limit within 1–2 years before RRT start. A European and an American study have found that general practitioners were less responsible for late referral than non-nephrology medical specialists [25,26].

The quality of pre-RRT care was evaluated by the use of specific drugs to prevent CKD progression and modify complications as well as the use of a common nephrotoxic agent. The frequency of peritoneal dialysis as primary RRT modality, arteriovenous vascular access in haemodialysis patients and renal transplantation waiting-list status also reflect pre-RRT care. It was found that fewer late referrals received RAS blocking agents or the D-vitamin analogue alfacalcidol. The use of short-term NSAID was the same in both referral groups but long-term use was a little higher in late referrals probably reflecting that both groups had significant proportions of patients with chronic pain conditions. Furthermore, late referrals less often commenced RRT with peritoneal dialysis, had an arteriovenous fistula or were on the renal transplantation waiting-list. Previous studies have shown similar results [1-5,7,9,12].

The late referred patients were characterized by older age and more comorbidity. This has also been reported in previous studies [5,7,9]. Acute decrease in renal function in old patients with moderate CKD might be a cause. In addition, general practitioners and non-nephrologists might consider old patients with CKD and comorbidity unfit for RRT. Subsequently, when old patients are hospitalized because of severe uremic symptoms, RRT is initiated. However, some studies have found no age differences between early and late referrals [1,25]. The different findings might be explained by type 2 error because of small study population size.

In accordance with our findings, previous studies have observed more cancer and cardiovascular disease in late compared to early referrals [7,10,25,26]. We used administrative registry data on hospital admissions because of myocardial infarction, stroke or cancer to study comorbidity. Probably, the overall cardiovascular comorbidity in both early and late referrals is underestimated because mild symptoms are treated in general practice.

As reported in other studies [7,12,25], we found that the renal diagnoses diabetic nephropathy, adult polycystic kidney disease and chronic glomerulonephritis were less frequent in late compared to early referrals. Non-nephrology physicians might be more aware of referring patients with well-known renal diagnoses.

Income and education was similar in early and late referrals in our study like in a French study [27]. Generally, persons with low income or short education are as frequent users of the publicly funded health care system in Denmark as persons with higher income and longer education [28]. In contrast, socially deprived patients in UK and patients with shorter schooling in France, Italy and Switzerland were more often referred late [25,29].

In accordance with our findings, one-year mortality adjusted for comorbidity was higher in late compared to early referrals [7,12]. This was also true when excluding lead-time bias [30]. In one study, no difference in one-year survival was found [10]. However, late referrals were younger than early referrals and analyses were adjusted for albuminaemia which has been shown to be a strong predictor of death and associated with late referral in incident dialysis patients [10,31].

The nationwide administrative and clinical registries used in this study offer complete sources of data as all citizens have free access to the tax-financed health care system. The validity of data including the date of RRT start in and completeness of the Danish Nephrology Registry is good [32]. Also, information in the National Patient Registry is assumedly valid and gives the possibility of complete follow-up [15-17], thus, the risk of misclassification bias of late or early referrals should be small.

Different definitions of late referral have been used in the literature: 1, 3, 4 or 6 month or level of serum creatinine [1,2,5,7,9,10,12,25]. The time needed to prepare for chronic dialysis is at least 3–4 months.

We only had information on plasma creatinine before RRT start in a subgroup of patients. Thus, the generalisability of the results depends on similarities of CKD progression in these patients compared to other patients. Late nephrology referral is of course unavoidable for patients with irreversible acute renal failure or rapidly progressive renal failure. Irreversible acute renal failure has been reported to constitute 11–38% of late referrals [1,10].

The retrospective design of this study only sheds light on the courses of patients starting active treatment of renal failure and not the ones receiving conservative treatment as we have no information on non-referrals. A prospective study, identifying persons with CKD by plasma creatinine, would provide additional information.

## Conclusion

Late nephrology referrals were well-known to the healthcare system before referral to renal units and more often had near normal levels of plasma creatinine within two years before RRT start. They infrequently received available treatment or optimal first RRT modality. More attention on referral of CKD patients from general practice and non-nephrology hospital departments to renal units is recommended.

## Competing interest

No competing interests to declare.

## Authors’ contributions

AK, MM and KH contributed to conception, design and acquisition of data as well as analysis and interpretation of data. KH drafted the manuscript. AK, MM and KH were all involved in revising the manuscript critically for important intellectual content and have given final approval of the version to be published.

## Pre-publication history

The pre-publication history for this paper can be accessed here:

http://www.biomedcentral.com/1471-2369/13/108/prepub
